# Designing an SMS reminder intervention to improve vaccination uptake in Northern Nigeria: a qualitative study

**DOI:** 10.1186/s12913-021-06728-2

**Published:** 2021-08-20

**Authors:** Chisom Obi-Jeff, Cristina Garcia, Obinna Onuoha, Funmi Adewumi, Winnie David, Tobi Bamiduro, Abdulrasheed Bello Aliyu, Alain Labrique, Chizoba Wonodi

**Affiliations:** 1 Department of Research, Direct Consulting and Logistics Limited, Abuja, Federal Capital Territory, Nigeria; 2 Department of International Health, International Vaccine Access Center, Johns Hopkins Bloomberg School of Public Health Baltimore, MD Baltimore, USA; 3 Department of Primary Health Care System Development, Kebbi State Primary Health Care Development Agency, Birnin Kebbi, Kebbi State Nigeria; 4 Department of International Health and Department of Epidemiology, Johns Hopkins Bloomberg School of Public Health Baltimore, MD Baltimore, USA

**Keywords:** Immunization, Vaccination, SMS Reminders, Text messages, Barriers, Mobile phone use, mHealth, Formative studies, Nigeria

## Abstract

**Background:**

Penta3 coverage in Nigeria was low at 33 % in 2017. The most reported reason for non-vaccination was lack of knowledge about the immunization place, time, and need. To address knowledge gaps and improve vaccination uptake, we designed an Immunization Reminder and Information SMS System (IRISS) to educate and remind parents/caregivers about immunization using SMS. A formative study was conducted to understand the contextual and behavioural factors that would inform the IRISS intervention design and implementation.

**Methods:**

We conducted the study in four Local Government Areas (LGAs) of Kebbi State Nigeria in October 2018, amongst a diverse selection of participants. Data on social norms about vaccinations, barriers to immunization uptake, mobile phone use, SMS message testing, and willingness to accept SMS reminders were collected from focus group discussions (*N* = 11), in-depth interviews (*N* = 12), and key informant interviews (*N* = 13). In addition, we assessed 33 messages covering schedule reminders, normative, motivational, educational, and informative contents for clarity, comprehensibility, relevance, cultural appropriateness, and ability to motivate action among community members from Argungu and Fakai LGAs. All interviews were analyzed using a thematic analysis approach.

**Results:**

We interviewed 135 people, and 90 % were community members. While we found positive perceptions about immunizations among those interviewed, pockets of misconceptions existed among community members. Lack of awareness on the importance of vaccination was a consistent reason for under-vaccination across the LGAs. In addition, most community members do not own phones, could not read SMS messages, and were unaware of how to check/open text messages received. Despite concerns about low literacy levels and phone ownership, community members still saw a role in SMS reminders when phone owners receive messages. For instance, community leaders can disseminate said messages to community members through existing channels such as town announcers and religious gatherings. Therefore, the SMS becomes a source of information, with phone owners acting as a conduit to community dissemination mechanisms. We generally found the tested messages to be relevant, motivating, and culturally acceptable.

**Conclusions:**

SMS reminders have the potential to bridge the information gap in community awareness for vaccination, which can translate to improved immunization uptake. In rural communities with low literacy levels and phone ownership, immunization information can be disseminated when existing community leadership structures are engaged.

**Supplementary Information:**

The online version contains supplementary material available at 10.1186/s12913-021-06728-2.

## Background

Vaccines are among the most successful and cost-effective public health interventions today [1]. A decade of evidence shows that vaccines prevent illness, save lives, reduce poverty and household expenditure, improve childhood physical development, and enhance equity [2–8]. However, there are some considerable challenges in ensuring target populations receive the recommended vaccinations.

Routine Immunization (RI) in Nigeria is offered free of charge to children less than two-year-old at government hospitals, primary health centres (PHCs), mobile/outreach stations, and some private hospitals. The current RI schedule comprises eight vaccines targeted at ten vaccine-preventable diseases (Table 1). In Nigeria, where vaccine coverage lags behind national goals, the government and development partners have made considerable supply-side investments over the last five years. However, despite these investments, uptake and coverage of immunization services are still suboptimal.

**Table 1:** Nigeria Routine Immunization Schedule and diseases they prevent

Age	Vaccine	Targeted Vaccine-Preventable Disease
Birth	BCG, Oral Polio Vaccine (OPV) 0, Hepatitis B 0	Tuberculosis, Poliomyelitis and Hepatitis B
6 weeks	OPV1, Pentavalent 1, Pneumococcal Congugate Vaccine (PCV) 1	Poliomyelitis, Diphtheria, Tetanus, Pertussis, *Haemophilus influenza* b, Hepatitis B, and Pneumococcal diseases
10 weeks	OPV2, Pentavalent 2, PCV2	
14 weeks	OPV3, Pentavalent 3, PCV3, Inactivated Polio Vaccine	
9 months	Measles, Yellow fever, Meningitis A	Measles, Yellow fever, and Meningitis
15 months	Measles second dose	Measles

In 2016/2017, a national survey indicated that only 33 % of children aged 12–23 months received all three doses of the pentavalent vaccine (Penta3) nationally [9]. Additionally, about 40 % of one-year-olds had not received any vaccines from the RI program. The survey found that a lack of awareness (42 %) about the place, time, and need for immunization was the most frequently reported reason for non-vaccinations in Nigeria [9]. While the Nigeria RI program has recorded some progress with Penta3 coverage increasing from 33 % in 2016/2017 [9] to 50 % in 2018 [10], the immunization coverage rate still falls short of national and global targets [11]. In addition, coverage varies by region across the country, with Northern Nigeria having low coverage compared to the South [10]. This regional difference has been attributed to demand-side issues rather than supply-side factors [12], despite the government and partners' investments and polio campaign efforts in the last five years. Demand-related issues still included lack of awareness and vaccine hesitancy due to lack of information and knowledge about the RI program and the benefits of immunization, myths and rumors, religious beliefs, and other social and political factors [13, 14]. For instance, polio communication interventions focused on polio, increasing the knowledge about polio and RI lags. In addition, the social determinants of vaccine uptake, such as low maternal education and gender barriers which undermine women’s autonomy to seek care or vaccination services, are pronounced in Northern Nigeria [14]. These fundamental social determinants continue to have residual effects on vaccination uptake even when communication campaigns are deployed.

Demand-side interventions that address the lack of awareness issues and increases mothers’ and caregivers’ knowledge to utilize vaccination programs are essential in achieving high immunization coverage [15–17]. However, communication interventions to improve demand for childhood vaccination in Nigeria, such as health talks for parents/caregivers in their local health facility and media, e.g., radio jingles, seldom addressed the most relevant determinants of low vaccination uptake [18, 19]. Thus, effective communication strategies that can address concerns that prevent vaccination uptake, educate people about the benefits of immunization and, inform them where and when to get vaccinated are needed.

This study aims to bridge the vaccination knowledge gap among caregivers by delivering geographically targeted and personalized vaccine information and reminders via SMS to populations at high risk of non- and under-vaccination in Nigeria. Studies have shown different success levels in deploying SMS reminders to improve immunization uptake and coverage rates [20–22]. However, these studies were done in settings with relatively high baseline immunization coverage ranging from 74 to 82 %, where vaccination services are available and accessible [20–22]. In addition, SMS reminders’ applicability in the Nigerian context is unknown, as most are implemented in urban and sub-urban areas [23]. Therefore, there is a need to design an SMS reminder intervention that can be tested in low immunization coverage areas, including rural and remote communities.

The Immunization Reminder and Information System (IRISS) intervention was designed to deliver immunization information and reminders via SMS to bridge the knowledge gap and improve vaccination uptake. Besides, the dissemination of information on dates of scheduled vaccination visits, the type of vaccines to receive, and the follow-up on parents who miss vaccination appointments have been identified as a potentially effective strategy for attaining and maintaining high immunization coverage [24]. Evidence from the intervention will provide information on the effectiveness of SMS for increasing immunization uptake and whether SMS can be deployed together with other activities to improve immunization coverage. Information of this nature is especially critical to low-resource settings like Nigeria, where there is competition for limited health funds.

To inform the IRISS intervention design and implementation, a formative study was conducted in Kebbi State, North-West Nigeria, to understand the drivers of low immunization uptake and the programmatic and telephonic context where the intervention would be deployed. The objectives of the formative study were to:

1. Understand social norms that drive demand for vaccination;
2. Understand key barriers to immunization uptake;
3. Understand mobile phone use patterns in target communities;
4. Assess parent’s willingness to opt-in for SMS reminders; and
5. Test proposed SMS messages for readability (style, tone, and length), acceptability (content, linguistic, and cultural), and delivery (timing and frequency).

### The theoretical basis of the intervention

**Fig. 1:**
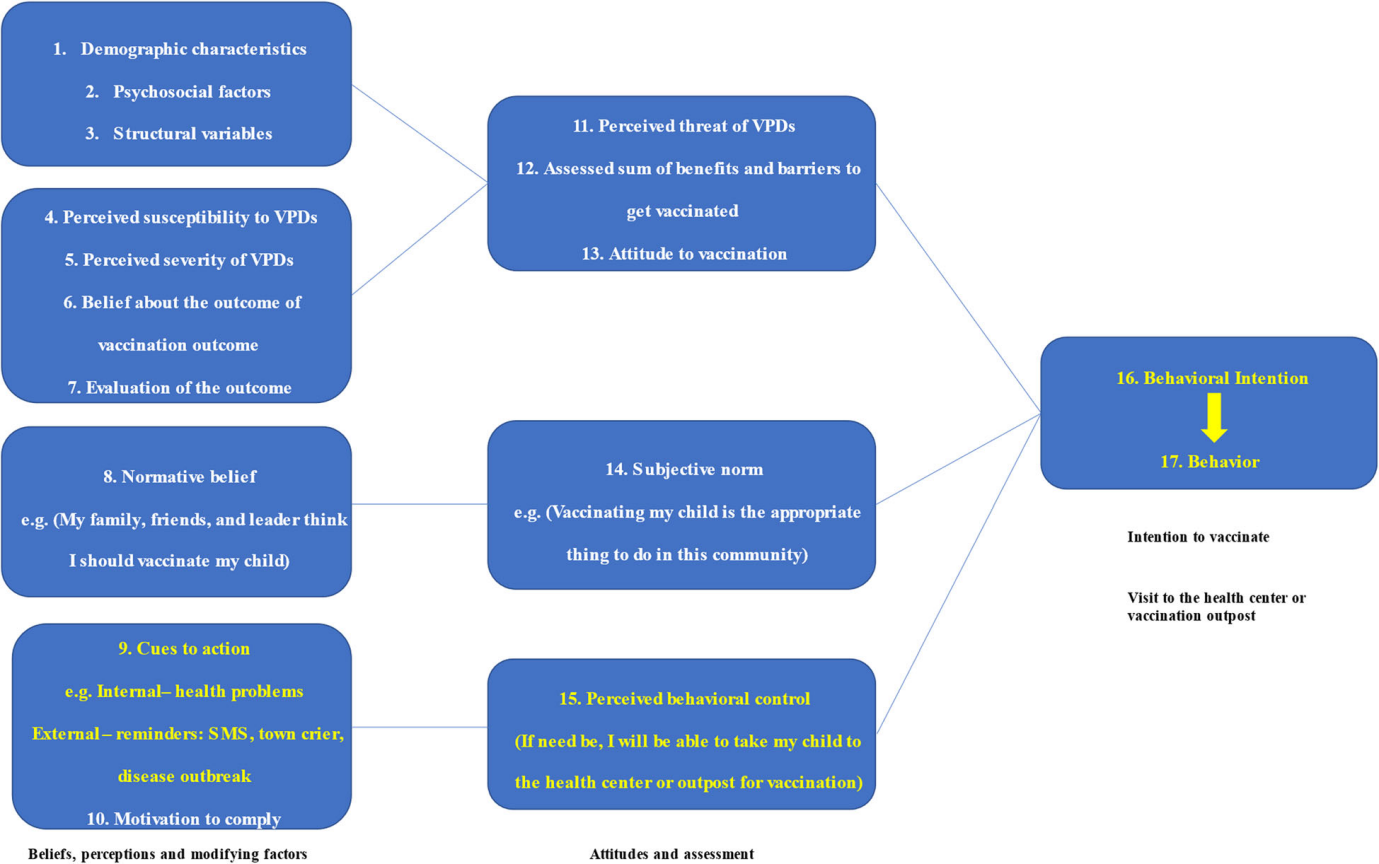
Adaptation of the health belief model and theory of planned behavior to explain vaccine uptake. We adopted these theoretical models from the concepts of the Health Belief Model and Theory of Planned Behavior. These models show that beliefs and perceptions can influence attitudes towards vaccination, which leads to an assessment of behaviors and influences behavioral intentions to vaccinate by ultimately visiting a health center. Elements highlighted in yellow represent the pathways of interest in the IRISS study

Evidence has shown that intent to vaccinate, community access, and facility readiness are key drivers of immunization coverage [25]. Our formative study was designed to influence vaccination by providing information on the benefits of immunization and reminders on vaccination schedules. This approach to increase intention to vaccinate was based on two theoretical models of health behavior – the Health Belief Model [26–28] and the Theory of Planned Behavior [29]. The Health Belief Model posits that the likelihood of adopting a health behavior is determined by individual perceptions (susceptibility to and severity of disease) and assessing the benefits and barriers of adopting the behavior. Beyond perceptions, individuals may need to be motivated or cued to action and believe in their ability to enact a behavior successfully. The Theory of Planned Behavior, on the other hand, suggests that an individual’s intention to engage in a behavior is determined by the individual’s attitude towards behavior, subjective norms, and perceived behavioral control. Figure 1 illustrates the adaptation of the two behavioral models.

### Intervention description

**Fig. 2:**
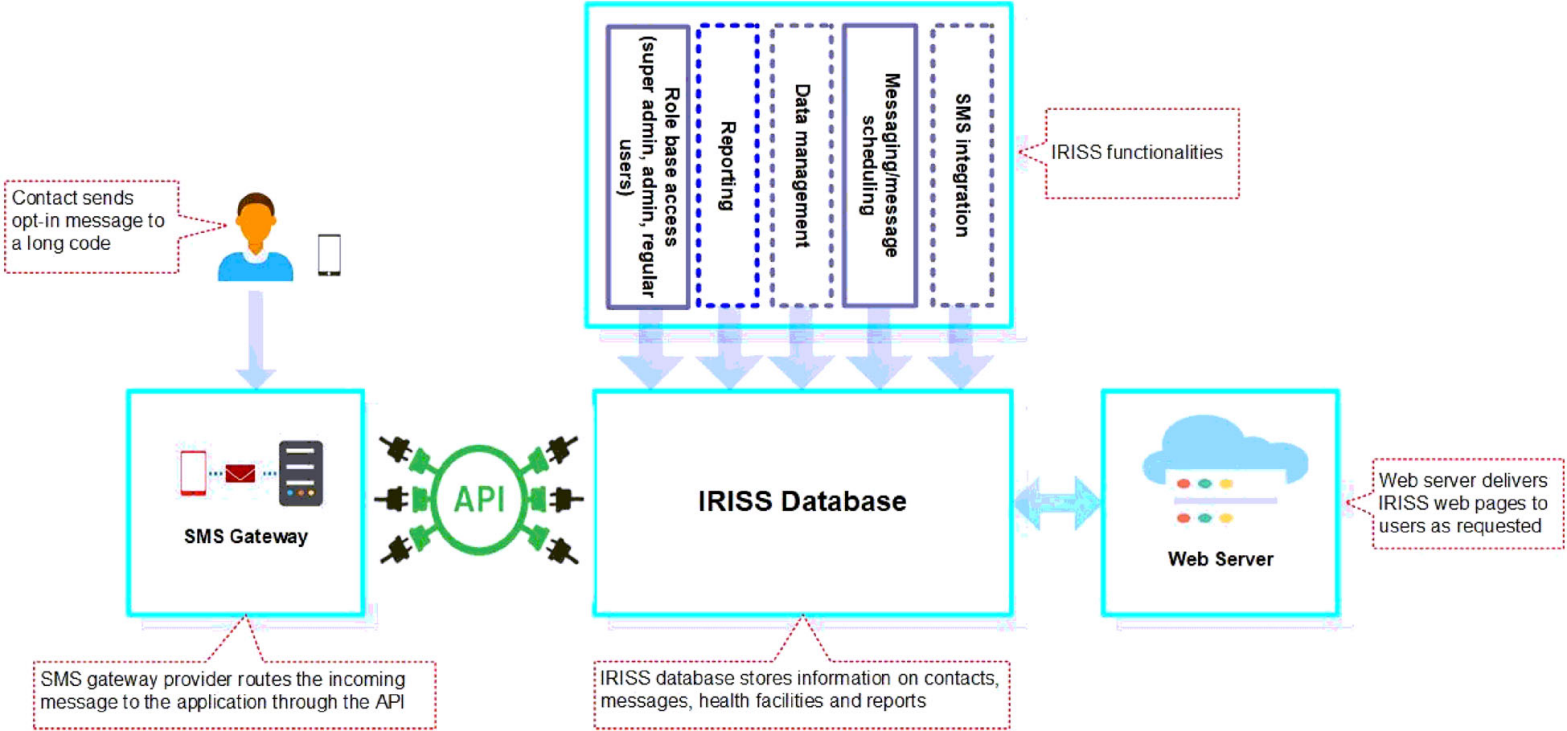
IRISS Architectural design. IRISS application was designed with an open-source framework using a hypertext pre-processor model view controller framework (Yii 2 framework) programming language. The design also includes a relational database of contacts, health facility immunization schedules, message library, and reports section. The application is integrated with an SMS gateway through an application programming interface to enable the system to send personalized and broadcast messages

The IRISS application is a cloud-hosted customized registration and message scheduling application for parents/caregivers and the public (Fig. 2). It was designed to provide automated registration and to deliver SMS messages in three ways:

1. Location-based SMS containing general information about immunization and advertisement to opt-in to the IRISS e-registry.
2. Location-based SMS containing information on the immunization days of the nearest health center(s) to individuals voluntarily opting-in to the IRISS e-registry (Leads); and
3. Individualized SMS containing information on the child’s vaccination schedule to parents/caregivers who voluntarily opt-in to receive personalized messages.

## Methods

### Study design

This was a qualitative study that involved focus group discussions (FGDs), in-depth interviews (IDIs) and key informant interviews (KIIs).

### Study setting

We selected Kebbi State for IRISS intervention implementation after a series of in-depth consultations and recommendations from the National Primary Health Care Development Agency and the National Emergency Routine Immunization Coordinating Center based on having: (i) the fourth-lowest RI coverage among the 18 low-performing priority states in Nigeria [9]; (ii) a high RI left-out rate (65 %) [9]; and (iii) the presence of strong political will for health.

Located in the northwest geopolitical zone of Nigeria, Kebbi has a total population of about 4.4 million and comprises 21 Local Government Areas (LGAs) and 225 political wards [30]. It is mainly rural, with only five LGAs considered urban or semi-urban. Kebbi's populace is predominantly Muslim, and LGAs are divided into four Emirate Councils: Argungu, Yauri, Zuru, and Gwandu, each with an Emir as the Supreme Leader.

Among individuals aged 15–24 years, only 31 and 42 % of women and men, respectively, were literate or attended secondary or higher education [31]. Despite the low literacy rate, it was encouraging to see that cell phone ownership was about 50 % [32]. Immunization activities in Kebbi are provided at government hospitals, PHCs, and mobile/outreach stations. However, most children (55 %) receive their vaccination from immunization campaigns such as yellow fever, measles, and polio campaigns compared to government hospitals (35 %), PHCs (32 %), and mobile/outreach stations (1 %) [9, 31]. Before the IRISS intervention study began in 2018, only 23 % of infants in Kebbi received BCG compared to 53 % nationally [9, 31]. Also, a mere 5 % received all recommended vaccines in the RI schedule compared to 23 % nationally [9, 31]. In addition, some supply-side challenges such as vaccines stock out at the health facility level and an insufficient number of and inequitable distribution of health workers did exist, contributing to sub-optimal RI performance in the State [13]. In recognition of the factors contributing to poor RI performance in the State, the National Emergency Routine Immunization Coordinating Center in 2017 identified Kebbi State as one of the 18 low performing states for subsequent RI intensification activities to increase immunization coverage. While some interventions have been implemented to address supply-side and demand-side issues, challenges exist in implementing these activities [13].

**Fig. 3:**
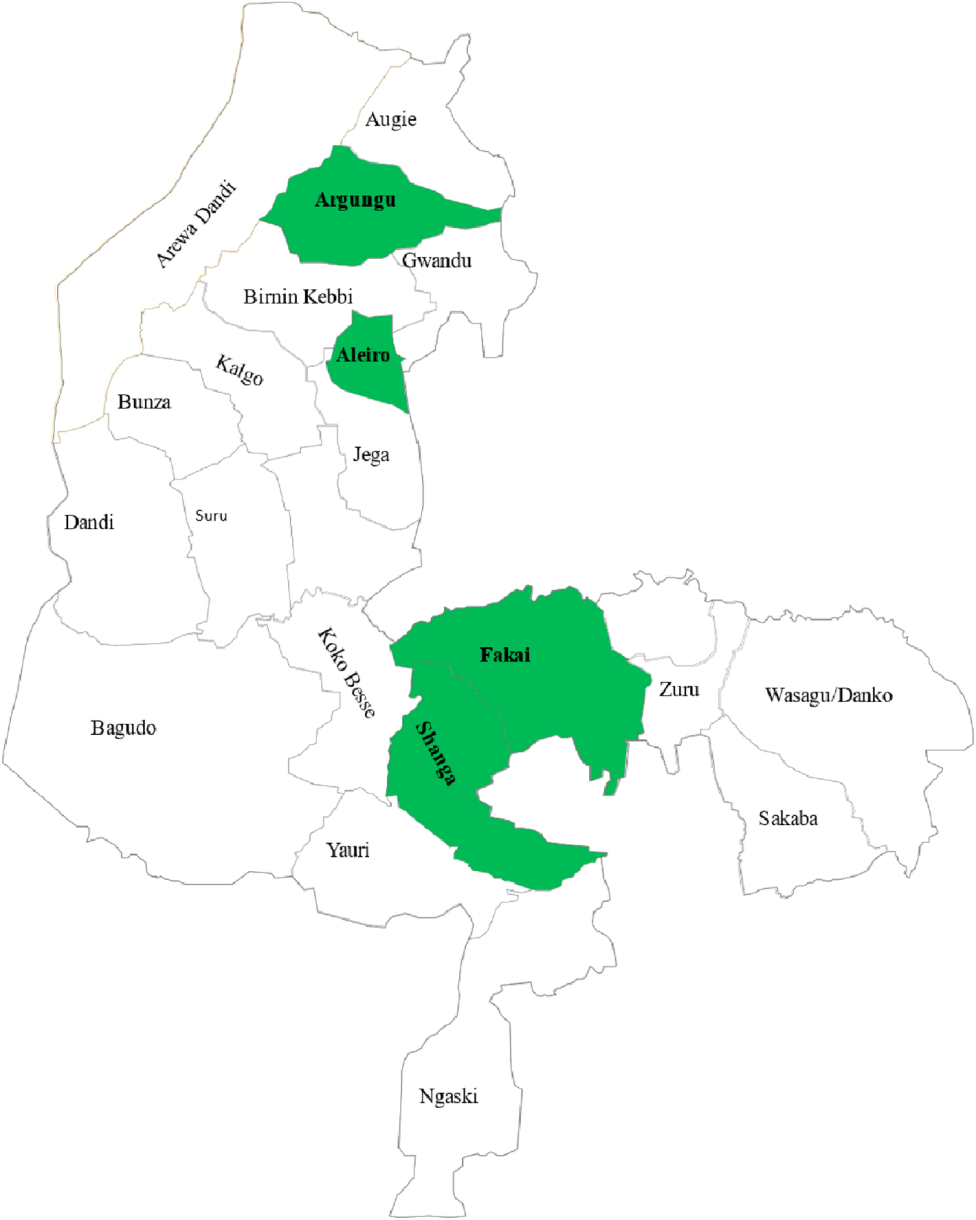
Map of Kebbi state showing the study LGAs Study LGAs are shaded green

Our formative study was conducted in four LGAs in Kebbi: Aleiro, Argungu, Fakai, and Shanga (Fig. 3).

These LGAs were selected to have balanced Emirate District representation, rural and urban classifications, and RI performance data from the 2018 third quarter (Q3) Lots Quality Assurance Sampling survey (Table 2) [33]. We selected these parameters to understand the different contexts and cultures in Kebbi, differences in mobile phone ownership and use, social norms that drive demand for vaccination, and identify key barriers to immunization uptake.

**Table 2:** Characteristics of formative study LGAs

S/N	Name of LGA	Emirate Council	Demography	RI Performance^1^	Phone Ownership^2^
1	Aleiro	Gwandu	Rural	23 %	45 %
2	Argungu	Argungu	Urban	10 %	42 %
3	Fakai	Zuru	Rural	35 %	5 %
4	Shanga	Yauri	Rural	53 %	3 %

^1^RI performance is defined as the proportion of children appropriately immunized for age assessed from the Q3 2018 Lots Quality Assurance Sampling survey; ^2^Phone ownership is defined as owning a phone or having access to a phone.

### Study participants

 FGDs and IDIs were conducted among community members, including parents of under-five children, parents of newborns, pregnant women, opinion leaders (traditional and religious leaders, persons of influence), Ward Development Committee (WDC) members, traditional birth attendants, young men and women, and male and female youth groups. In addition, participants for the KIIs included state-level policymakers, RI program managers at state and LGA levels, development partners, and RI providers in health facilities. We purposively selected the participants to ensure the representation of key constituencies of the target audience at all levels.

All community participants were eligible to be included if they were community residents for a minimum of one year and available on the scheduled interview date. Phone ownership was not required for participation. In addition, parents/caregivers participated in the interviews if they had under-five children; and were between the ages of 25 and 35 for the women and 25 and 45 for the men. The young men and women are those aged 15 to 24 years. Policymakers, RI managers, and providers were eligible if they had a minimum of one year of experience in their current role. The FGD participants were identified and recruited at the settlement level through their community leaders (District Heads and *Mai-unguwas*) following sensitization about the study to these leaders. While recruiting discussants for the FGDs, we identified individuals for the IDIs whom community leaders and LGA program managers suggested as eligible individuals interested in participating in the study. These individuals were contacted by phone for recruitment at least one day before the interview to confirm their interest and availability to participate in the interview. Furthermore, we identified and selected our KII participants through the study advisory group, the State Primary Health Care Development Agency, the Kebbi State Emergency Routine Immunization Coordinating Center, and the LGA program managers.

A total of 11 FGDs, 12 IDIs, and 13 KIIs were conducted among 135 people (see Additional file 1 for the full list of participants interviewed and type of interview conducted).

### Data collection

FGDs, IDIs, and KIIs were conducted between October 15 and 19, 2018, using semi-structured interview guides. Interview guides were designed based on the study aim and objectives and were pre-tested in Jega LGA to ensure we correctly framed all questions.

Data collectors trained in qualitative research methods were responsible for conducting the interviews, the study team tracked all interviews, and external personnel transcribed them. Before participation in interviews, oral consent was obtained from each participant using a standard verbal consent script that highlighted identity and confidentiality protection. Where necessary, we obtained consent in Hausa, the most common local language.

We collected socio-demographic information such as gender, age, educational status, and the participant type from all interviewed using a cover sheet (see Additional file 2). In addition, all interviews conducted were tracked using a tracking sheet (see Additional file 3). Interviews and discussions lasted an average of 45 min and were conducted either at the participant’s office, home, or a convenient location within the community.

We developed the interview guides in English, but most interviews (30) held at the community level were conducted in Hausa. Interviews conducted in Hausa were transcribed in Hausa and translated to English before analysis.

### Message testing

A total of 87 messages covering normative, schedule reminder, motivational, educational, and informative contents were developed for testing based on a literature review of the barriers to immunization uptake in Nigeria [34–43]. These messages were translated into Hausa by students from the University of Jos, Plateau State, Department of Linguistics, and validated by IRISS project officers for accuracy. Subsequently, Health Communication Officers from Kebbi state reviewed the translated messages for context relevance, where 33 messages were useful for testing (Table 3). See Additional file 4 for messages in Hausa. Thus, a two-step process was adopted to validate the messages. The 33 messages were tested among 57 community members from Argungu (*N* = 21) and Fakai (*N* = 36) LGAs for clarity, comprehensibility, relevance, cultural appropriateness, and ability to motivate action.

**Table 3:** IRISS Messages in English

#	Content	Messages in English
1	**Normative messages**	Are you aware that your neighbour has taken his/her child for vaccination? What are you waiting for, don’t be left behind
2		Did you know that Emir … supports vaccination? He encourages us to be good parents for our children and get them vaccinated for better health
3		Husbands allow your wives to take our children for vaccination
4		Our religious leaders have immunized their children. What are we waiting for?
5		God has given us the responsibility to take care of our children. We should make sure to vaccinate them
6		Dear Parents, we need to make out time and get our children vaccinated. That time will save their lives
7	**Scheduled reminder messages**	Remember to keep your vaccination card safe! It will guide you for the next vaccination visit at *Gafara* hospital
8		Don’t forget! It takes 5 visits to the hospital to complete your child’s vaccination: at birth, 6 weeks, 10 weeks, 14 weeks and 9 months
9		There will be a free vaccination outreach at the Chief’s Palace tomorrow. Bring your child to receive vaccines. Remember, vaccines save lives!
10		*Ribah* clinic is holding a vaccination session tomorrow from 8 am to 2 pm. Please take your child there to vaccinate them and encourage your neighbors to bring their children.
11	**Motivational messages Motivating**	Guess what? These are 3 top reasons why you should vaccinate your child: (1) vaccines prevent diseases (2) vaccines save lives (3) vaccines are safe
12		Each visit to the health centre for vaccination will reap many health benefits. Say YES to good health, be on time and complete your child’s vaccination
13		Get your baby vaccinated. Even though he/she cries after the injection, they will be very happy in the future when they become healthy and can support you
14		Dear parents, aren’t you happy when your babies greet you with smiles after you come home from work? Make them happier by vaccinating them against diseases
15		Bearing children and watching them grow into healthy adults is an exciting experience. Give them a healthy start to life by ensuring they are fully vaccinated
16		All childhood vaccines given in government health centers are free. Yes, FREE. Don’t miss this opportunity to protect your children at no cost
17		Everyone should be a champion for immunization. Wherever you are, encourage your neighbours to vaccinate their babies. You are doing a good deed in saving lives
18		Promoting the health benefits of vaccines is a collective effort. Do your part by telling relatives, neighbours, and friends to vaccinate their children
19		To ensure that every child in this community receives all the basic and necessary vaccines they need, we must spread the word that vaccines work
20		Please share information about vaccination in your community. You can start by telling that new mother in your area that vaccines work and saves babies lives
21	**Educational messages**	The first vaccination that your baby will receive are **BCG, Hep B** and **OPV0** and it should be taken within the first 2weeks of birth.
22		The second vaccination that your baby will receive are **Penta1, PCV1** and **OPV1** and it should be taken at 6 weeks of age.
23		Have you heard of Penta vaccine? It protects against 5 diseases: they are diphtheria, whooping cough, tetanus, HiB and Hepatitis B
24		The third vaccination that your baby will receive are **Penta2, PCV2** and **OPV2** and it should be taken at 10 weeks of age.
25		The fourth vaccination that your baby will receive are **Penta3, PCV3, OPV3** and **IPV** and it should be taken at 14 weeks of age.
26		The fifth vaccination that your baby will receive are **Measles** and **Yellow Fever**, and it should be taken at 9months of age.
27		It is **your right** to have free and safe vaccination at all government hospitals. Go and let your child receive their vaccination in the hospital closest to you
28	**Informative messages**	Having a fever or swelling in the injection area after vaccination is normal. If the baby gets worse, quickly take the child to the hospital for proper care
29		Vaccines do not cause sickness. Vaccines protect babies and little children against germs which cause diseases
30		All vaccines given in Nigeria’s vaccination schedule are confirmed by the government to ensure safety before they are given to children
31		A child can still get vaccinated if they have a mild illness or fever or are taking antibiotics. But contact your health provider if you have any questions
32		Giving a child more than one vaccine at a time does not affect the child. Instead, it protects the child from many germs that cause diseases
33		Getting more than one vaccine at the same time does not harm a child. It is very important to receive all vaccine doses for full protection completely

 We conducted the message testing using an interviewer-administered questionnaire that was individually assessed by participants, followed by a group discussion. Participants, including groups of opinion leaders and parents of children under five, were divided into groups, and each group was assigned a facilitator and a recorder. Messages were read one after the other by the facilitator to the group. We provided each participant with an answer sheet (paper-based). They individually assessed the messages to ascertain whether they were clear, relevant, motivating, culturally appropriate, or had difficult words/phrases. Participants individually rated each question “Yes” or “No” separately for the following attributes: clarity, comprehensibility, relevance, cultural appropriateness, and ability to motivate action. The ratings for each question were then collated by attribute. Any question that was assessed by the majority to be unclear, not comprehensible, not relevant, not culturally appropriate, and not able to motivate action was flagged for discussion by the group. During the group discussion, questions were revised or rephrased to participant’s satisfaction.

#### Data analysis

All interviews were audio-recorded, transcribed verbatim, and analyzed using a thematic analysis approach. COJ and CW reviewed all transcripts and inductively generated codes relevant to the study aim and objectives. We assessed the codes for internal homogeneity and external heterogeneity to form appropriate themes. This assessment ensured that the data within the themes connected meaningfully together while presenting a clear distinction between the themes. COJ entered the transcripts, codes, and themes into Atlas.ti® software for analysis. COJ coded each transcript and queried the data for meaningful content using the Atlas.ti® software Query tool. COJ and CW interpreted it based on identified themes related to the study’s aim and objectives. Social norms were analyzed using Berkowitz’s social norms theory framework [44], adopted in a similar immunization communications study in Northern Nigeria [45]. We explored the social norms divided into personal, injunctive, and descriptive norms (Table 4). All themes were analyzed by respondent type and LGA. We used exemplar statements for illustrative purposes, but quotes were anonymized to protect participants’ confidentiality. The completed message testing questionnaire and interview tracking sheets were entered into Microsoft Excel® and analyzed for frequencies.

**Table 4:** Description of Personal, Injunctive and Descriptive norms

Category	Description
**Personal norms**	These norms are personal beliefs and feelings regarding vaccination and RI services provided by health workers in our study settings. It relates to attitude towards vaccination and personal decision about vaccinating or not vaccinating their children.
**Injunctive norms**	This is an assessment by people interviewed of how much others approve or disapprove, encourage or discourage vaccination.
**Descriptive norms**	This explains how most people’s beliefs and behavior, i.e., behavior of others regarding vaccination in our study setting, encourage or discourage vaccination.

## Results

### Socio-demographic characteristics of participants

We interviewed 135 people, of which 90 % were community members and 62 % were male. Participants’ age ranged from 19 to 80 years, with an average mean age of 38 years (standard deviation ± 14.4). More than half of our participants had formal education (64 %), while about 30 % attained secondary school or higher (Table 5).

**Table 5:** Socio-demographic characteristics of participants

Characteristics	Number (*n* = 135)	Percentage (%)
***Sex***		
Male	84	62 %
Female	51	38 %
***Attended formal education***		
Yes	86	64 %
No	49	36 %
***Highest level of formal education completed***		
None	49	36 %
Primary School	19	14 %
Secondary School	40	30 %
Graduate	25	19 %
Post Graduate	2	1 %
***Respondent type***		
Community members	122	90 %
Program managers	6	4 %
Health workers	6	4 %
Policymaker	1	1 %

### Social norms about vaccination

#### Personal norms about vaccination

All participants interviewed in the four LGAs believed that vaccinations are important for the child’s wellbeing, having witnessed its benefits. From the interviews with community members, including parents, we found a positive shift in vaccination perceptions from a previous negative belief and rejection of vaccination. This positive shift had influenced their intention to vaccinate, even among those without children. Also, vaccinated children were perceived to be healthier than those who were not and were at-risk during outbreaks. Finally, there was a general confirmation that no religious belief or traditional/cultural practice forbade vaccination uptake across the four study settings.

*“RI is very important. I am saying so because I have experience living in an extended family house. I wasn’t in support of RI, so I forbid my family from doing it. But there was an outbreak one day, and it affected all my children, and the other kids that took RI were safe; then I realized how I wronged my family by not protecting them from diseases. Since then, my family are always at the front in RI activities”.* (Father of under-five)

Additionally, the community members reported they had complete trust in their health workers, the information they provide, and the services they render. For example, most community members reported satisfaction with the information health workers provided on managing vaccination side effects in children.

*“We are satisfied with the services they are rendering because they use to explain to us about what to expect after our children receive their immunization and how to manage them”.* (Mother of under-five)

Interestingly, we found little concern about adverse events following immunization (AEFI) among all community participants interviewed.

*“Yes, we do have such cases, but we didn’t take that to be a problem since they’ve already told us that RI can sometimes give little reactions such as fever. So, they give paracetamol for that, and it helped send the fever away”.* (Father of under-five)

#### Injunctive norms about vaccination

We discovered that traditional and religious leaders wielded significant social influence and were a strong force in promoting vaccination in communities. Their trust and approval of vaccination translated into ultimate acceptability in the community.

*“What encourages us to take our children for immunization is how our religious leaders give a sermon and tell us about the benefit of vaccination and assurance that this immunization does not have side effects. Once the District Head and Imam come to the mosque and give a sermon, and command us, I feel much at rest to take my child for immunization”.* (Influential person)

However, some community members reported that while some religious leaders promote vaccination, others perceive receiving vaccination as not believing in God’s utmost protection. As a result, these said groups of religious leaders had negative attitudes towards vaccination uptake and other health services.

Despite the general assertion that vaccination was important, there were still pockets of misconceptions. These misconceptions were related to community members’ beliefs that too much focus is placed on vaccination at the expense of other community needs/issues, vaccines are unsafe and cause sterility, and other personal or religious beliefs. The misconceptions mentioned above were attributed to a lack of knowledge and ignorance about the benefits of RI.

**Participant 1**: *Some people don’t believe in immunization simply because their parents didn’t take it during their time, and they are still okay. ***Participant 2**: *Some of them believe the immunization antigen they bring to us is already expired. ***Participant 3**: *Some people believe that immunization antigens kill their eggs*. (Mothers of under-five)

#### Descriptive norms about vaccination

We found that behaviors of relevant others influenced vaccination uptake in the study settings. For example, community leaders used town announcers to inform community members about immunization days. They also mobilized community volunteers to educate community members on vaccination benefits to encourage uptake and resolved non-compliance issues and sanctioned defaulters. On another note, some community leaders desired to be informed and involved in vaccination-related activities by health workers.

*“At times, the RI workers do not announce to the community when they are coming. Had it been, they informed us as community leaders, most of the problems will not arise. We, the community leaders, need to be part of the RI work, which will make it easy for our people to co-operate”.* (WDC member)

Gender roles also played a crucial role in informing decisions for vaccinations. For example, while there was general acceptability and support from fathers in Aleiro, Argungu, and Fakai LGAs for vaccinating newborns, some fathers in Shanga LGA did not approve of newborns being vaccinated.

*“I truly can’t give my 2-weeks baby to be vaccinated because I believe he is not strong enough for that, but I can take him myself if he got 2 months older. Then I will have more confidence”.* (Father of under-five)

Furthermore, we learned that some community members do not perceive vaccination as important.

*“One key thing I’ve observed is that here, they have not yet seen it as an obligation to go and take immunization. It’s still a lot of “we want you to come and take immunization”. They haven’t yet seen it as “I need to go and take immunization.”* (Development partner).

### Barriers to immunization uptake

**Fig. 4:**
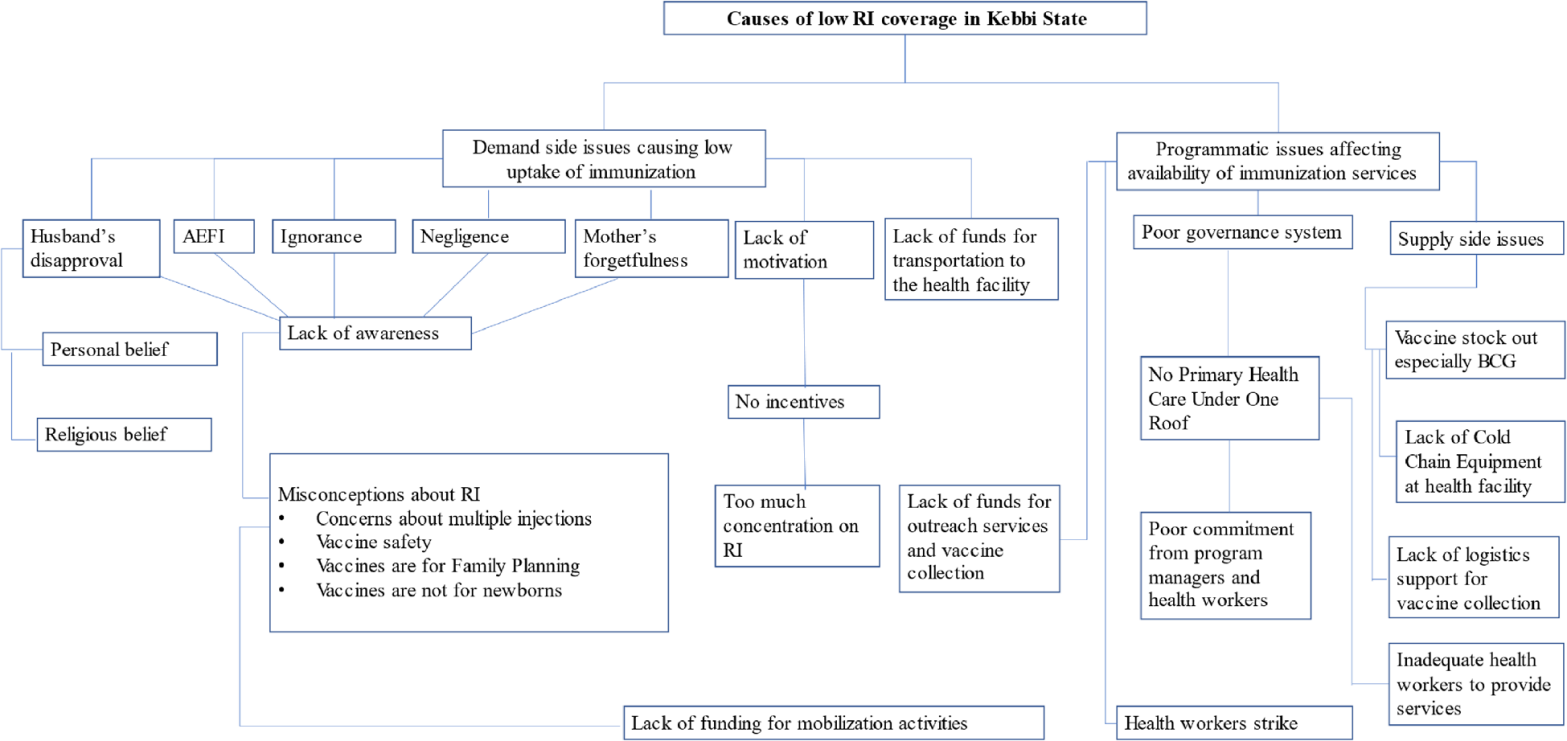
Thematic map of the causes of low RI coverage in Kebbi State. Causes of low RI coverage in Kebbi State can be divided into demand-side issues and programmatic-side issues. Lack of awareness of immunization’s importance was a major contributor to poor uptake, while inadequate funding and other programmatic issues contributed largely to the unavailability of immunization services

Key barriers to immunization uptake and availability of immunization services identified in the study settings are listed in Fig. 4. Ignorance of the benefit of vaccination and AEFI, specifically Penta vaccination pain and fever, were the most common barriers to vaccination uptake in all the LGAs. These two barriers were the primary reasons for poor vaccine initiation with the BCG vaccine and low completion of the three Penta vaccine doses. In addition, while almost every community member interviewed knew how to manage AEFI cases, some parents did not continue subsequent vaccinations due to the associated pain and discomfort. Participants attributed these two barriers to a lack of awareness of RI in general.

*“Yes, some of the villagers do not complete the immunization because when they did vaccinate, and their children’s leg swell or they got a fever, they will not go for the next immunization due to lack of the knowledge of its important”.* (Young women)

Probing further on community awareness of RI, we found that most RI information is received from town announcers who mainly publicize immunization days but do not educate community members on the importance or benefits of RI. While most participants knew about the benefits of vaccination, the majority lacked knowledge about the complete RI schedule and only learned about vaccines against polio and measles. Meanwhile, community members expressed the need for incentives to motivate them to vaccinate their children.

Participants from Aleiro, Argungu, and Fakai LGAs also reported other barriers such as lack of funds for transportation to vaccination center and negligence or mothers forgetting.

From the RI provider perspective, low awareness of RI is further complicated by limited social mobilization activities, attributed to a lack of funds. RI providers believed most RI activities were inadequately funded, especially logistics support for outreach sessions and vaccine collection.

“*The reason is lack of awareness to the community and lack of support to the RI providers by the government. The RI providers have to use their money to pay for transport while the government is not paying enough salary*”. (Health worker)

Other programmatic issues affecting the availability of immunization services and RI performance include supply-side problems, poor governance, and commitment from providers and health workers (Fig. 4).

### Mobile phone use pattern

Mobile phone ownership in the study communities was low, especially among those in rural areas, and the majority of phone owners were husbands/fathers who could not read SMS messages.

 However, most participants who own a phone but cannot read indicated willingness to ask someone to read the SMS messages.

“*Some of us cannot read, but we can give it to someone to read to us*”. (Mother of under-five)

Mobile phone behaviors also varied among participants. While some acknowledged that they view their phones when they get SMS messages and returned missed calls, others primarily used their phones to receive calls and were not aware when they received SMS messages or ignored them. Other participants indicated that some people switch off their phones or hang up when called by unknown numbers.

*“I am telling you, most people do not use to view the messages sent to them. There is a man that I checked his phone; he has over 100 unread messages, so sending a message will not be a helping matter. And this man, when his phone ring, checked and did not know the number or did not recognize the voice he switched off the phone immediately or drop the call.* (Opinion leader)

Meanwhile, paying attention to one’s mobile phone or SMS messages was associated with the person’s educational and employment status; and if he/she was expecting an SMS message.

*Civil servants are the ones that pay attention to their SMS because of their work. The women at home who are not working don’t care about SMS. Also, not every woman has a phone.* (RI provider)

### IRISS design and implementation

 We assessed participant’s perception on several attributes: (i) the IRISS intervention and SMS reminders; (ii) acceptability of the intervention; (iii) preferred person to receive SMS reminders from and the preferred mode of communication, language, frequency, and delivery time; (iv) willingness to sign-in for SMS reminders; (v) potential challenges; and (vi) strategies to address those challenges.

All participants perceived IRISS as a timely intervention that will promote RI demand, especially help remind those who forget their child’s immunization sessions and educate parents on the importance of RI.

*“This is really a good idea, which will remind those who easily forget their RI scheduled date, and it will all encourage others to see the importance of RI.”* (Opinion leader).

We identified no cultural and religious objections to receiving SMS reminders in all study LGAs. If received, the participants indicated a willingness to disseminate SMS messages to community members without phones.

“*My advice is to send the message, but it should be in time before Juma’at prayers so that I can deliver the message after or before prayer. But you should send the message in Hausa saying this, and this will take place, then you leave us, we know how to handle it. The problem we are having here is a lack of information, and when we get the information, there will be no problem. And we will spread the news.”* (Opinion leaders).

Most participants preferred to receive SMS reminders from their traditional leaders because they are well-respected, while few preferred health workers because it is health-related.

“*Through the traditional leaders, because they can send the message to all the town member that this message has arrived.*” (Parent of under-five).

“*Information spread by the health personnel is the best because it is a health-related issue*” (Pregnant woman).

Again, some mothers suggested sending SMS reminders to their husbands whose consent was needed before a child received immunization. They stressed this to be important as most husbands own mobile phones, and their permission will encourage more opt-in for SMS reminders.

*“It is better if you can send the message to our husbands because they are the ones who take things seriously and give permission to the mothers to take their children for immunization”*. (Mother of under-five)

There were similarities in how people preferred to receive immunization SMS reminders and messages. Among 29 community members and health workers, 52 % preferred phone/voice calls, while 48 % reported SMS messages preference. Those that chose phone/voice calls cited reasons around most people’s low literacy level and may not notice when they receive SMS. In contrast, those who preferred text messages said it would serve as a reminder, unlike phone calls, that people will quickly forget, and most people do not pick unknown numbers. However, to ensure all community members have an equal chance of getting the messages, participants suggested using text messages and town announcers.

*I suggest using both ways, the text and the town announcers, because some of us are so busy that we can easily get carried away by our day-to-day activities, but the text can be a reminder*. (Fathers of under-five)

While most participants preferred to receive the SMS messages in Hausa a day before the immunization session, there were differences in preferred delivery time. Most preferred the early hours of the morning, while others preferred late evenings.

Furthermore, all community members indicated a willingness to opt-in for SMS reminders. Similarly, health workers were happy to help register mothers during fixed and outreach vaccination sessions.

There were concerns about low mobile phone ownership and network connectivity, especially in remote areas. Likewise, the inability to read text messages due to the State’s low literacy level was challenging.

“*Truly, we don’t have a phone, and we don’t know how to read even if you send us a message, we cannot read it, but if you message our husband, they will inform us, but we cannot say if they would be able to reply you*”. (Mother of under-five)

On promoting the IRISS intervention, participants recommended engaging community leaders and conducting sensitization activities in communities before the commencement of the intervention.

*I want to add on the third one, which is to inform the district head, but still, you have to enlighten the people on the importance of the message and the immunization so that when they receive the message, they will know how important the message is*.” (Opinion leader).

Program managers suggested designing the intervention as a “status symbol” by sending reminders to individuals already accessing the services to encourage more registrations. The broadcast messages should be kept short and simple and include time, date, venue, and RI importance. Health workers should also be engaged to register newborns because of their literacy level rather than traditional birth attendants and traditional barbers.

### Message acceptability

We generally found the tested messages in Table 3 to be relevant, motivating, and culturally acceptable in Fakai LGA. However, 6 % of participants felt normative message #1 was unclear, while 5 % felt the same message had a problematic word/phrase. During the FGD, fathers felt the message had a bit of advice and question that could be confusing. They suggested including the benefits of immunization to prompt action.

Participants in Argungu LGA felt normative message #6 was irrelevant (52 %), discouraging (52 %), culturally unacceptable (52 %), obscure (24 %), and had difficult words/phrases (24 %). Furthermore, they judged schedule reminder message #8 to contain a difficult word/phrase (5 %); #9 was culturally unacceptable (52 %), and motivational message #11 was also socially unacceptable (52 %).

 Additionally, participants read text messages sent to their phones and attempted registering into IRISS using a format provided. However, typos and date formatting issues were common. Due to these texting errors, we could not complete the registration into IRISS without additional facilitators’ guidance, and simplification of the registration process was needed.

## Discussion

Our study revealed that participants generally support immunization, showed a willingness to receive immunization SMS reminders and messages, and acknowledged community leaders’ influence to help promote the IRISS intervention in their communities. Nevertheless, our results revealed several vaccine uptake barriers consistent with other studies in Nigeria [38–41, 46–49]. This study identified a lack of awareness of vaccination’s importance as the major contributor to immunization uptake barriers across the study LGAs. Though the number of educated participants was slightly higher than those uneducated, the results show that not everyone fully understood the full range of benefits from vaccination and the diseases prevented through the RI schedule. These findings corroborate the evidence that low awareness and knowledge about RI affect immunization uptake and coverage rates [41]. This underscores the need to improve RI awareness and education and provide information on vaccine services at health facilities, which the IRISS intervention aims to achieve through SMS reminders. Despite the general belief that vaccination was important, the role of community members’ consistent education cannot be overemphasized. Indeed, evidence has shown that children are more likely to get vaccinated if their parents understand vaccine-preventable diseases, vaccination benefits, and the RI schedule [41, 50].

We also found that inadequate funding and other programmatic issues contributed to the unavailability of immunization services and low vaccine coverage in the State. Availability and accessibility of vaccination services are key to improving immunization coverage [25, 51]. Therefore, policymakers and program managers should promote best practices by ensuring the availability of funds through basket funding to support outreach and vaccination sensitization activities and instituting a strong accountability mechanism that holds all stakeholders responsible for the poor implementation of RI programs.

While those interviewed perceived vaccination as important and beneficial to a child’s wellbeing, some religious leaders and husbands in the community did not support vaccination due to religious and personal beliefs. It was evident that such beliefs could contribute to the low uptake of vaccination services. Several studies have shown that sociocultural norms and beliefs exert a powerful influence on an individuals’ attitude and decision towards vaccination and how they react to health promotion messages and interventions [42, 52–56]. Thus, it is pertinent to ensure that religious leaders and husbands understand and promote the benefits of immunization, considering their strong influence in decision-making in Northern Nigeria [57]. Including men in this formative study was done, in part, to address their resistance to vaccination and build support for mothers to vaccinate [58].

This study also revealed two key challenges to delivering an SMS-based intervention: low literacy levels and low phone ownership. Given the State’s low literacy level, it was not surprising that most community members cannot read SMS messages. Furthermore, among participants owning a phone, many did not use SMS messages as a standard means of communication, and some attested to not being aware when they received SMS messages. Regardless of these challenges, participants generally welcomed the use of SMS messaging through IRISS to bridge the knowledge gap on RI. Additionally, the willingness of mothers to vaccinate their children reflected the desire for immunization services.

 When discussing the IRISS intervention specifically, participants believed the IRISS strategy would be successful when combined with other message deployment methods as add-ons to the strategy. For example, sending messages to community gatekeepers such as the traditional leaders, religious leaders, WDC members, and community volunteers to disseminate the information using town announcers and community gatherings. Most notable was involving community leaders, who are well-respected in the communities, in disseminating SMS messages. Their endorsement and involvement in the intervention would ensure equitable distribution of the SMS messages even to those without phones. Community members also envisaged greater project acceptability and feasibility if both SMS delivery to the public and through town announcers – who are under the employ of traditional leaders – were used as RI message dissemination modes. In essence, using town announcers would benefit community members who do not own phones and those who cannot open/read SMS messages.

 Participants were willing to encourage other community members to check/read their SMS messages and seek assistance if they could not, which is an added benefit for the intervention. This also aligns well with participants’ preference to receive SMS reminders from health workers since the SMS messages were health-related. As trusted community members, health workers could educate and assist mothers in registering their newborns for personalized SMS messages based on the RI schedule.

We believe that introducing the IRISS mHealth intervention in these communities would serve as the needed impetus to improve RI awareness and knowledge and engender community capacity building in mobile SMS usage [59–61]. Additionally, it would strengthen health workers’ capacity in using mHealth technology [62–64]. Following the message testing results, we modified the SMS messages to include immunization benefits to prompt community members.

### Modification of IRISS intervention design

Based on these formative study results, we modified the IRISS intervention design to ensure its successful implementation.

1. We leveraged existing community institutions/structures such as community leaders and gatekeepers to promote IRISS intervention and health workers to facilitate IRISS registration.
2. We sent targeted IRISS broadcast messages to select individuals and community gatekeepers such as the traditional leaders, religious leaders, WDC members, and community volunteers to disseminate the messages.
3. We revised IRISS messages to include information dispelling misconceptions around vaccination and highlight the health implications of non-vaccination.
4. We conducted sensitization activities before deploying IRISS intervention to encourage people to open and read their text messages and seek assistance if they cannot read.
5. We encouraged community leaders to disseminate immunization messages received using town announcers and at community gatherings.
6. We simplified the registration process and included a step-by-step guide with examples on registering into IRISS in all information, education, and communication materials.
7. We advocated for the state policymakers to ensure adequate funding, implementation of RI activities, and services availability.

## Limitations

Given the study methodology, our analysis was based on the study participant’s responses, which was limited by their size. Thus, our findings may not be generalizable to all populations. Despite these limitations, there were several strengths. First, we ensured most participants were community members in the interviews since they were the project’s ultimate beneficiaries. We did this to provide a comprehensive and more grounded approach to inform the IRISS intervention design and implementation. Second, since men were the primary decision-makers in families in Northern Nigeria [65, 66], we ensured that a more significant proportion of those interviewed were males to facilitate decision-making to vaccinate.

Overall, we were able to discover perceptions about vaccination among other community members as well as cognitive (relating to the understanding of immunization), psychosocial (about lack of motivation to vaccinate), and practical (relating to access to vaccination) barriers to immunization in the community. We were also able to identify strategies for designing and implementing the IRISS intervention to achieve its goal.

## Conclusions

Lack of awareness on the importance of immunization was a consistent reason for incomplete vaccinations in Kebbi State, Nigeria. SMS reminders have the potential to bridge the vaccine information and education gap in community awareness, which could translate to improved uptake of immunization services. Our study findings have provided insight into the IRISS intervention design, including refinement of SMS messages, sensitizing community members before implementation, and SMS messages targeting community leaders. The involvement of community leaders and health workers would greatly support using an SMS-based intervention. While there were challenges with low phone ownership and poor literacy rates, sending the text messages to community leaders and using existing community structures to disseminate SMS messages offline can extend the intervention’s reach to caregivers who do not own phones or cannot read/write. Integrating the IRISS intervention with existing leadership community structures may create the needed awareness to drive RI demand and uptake.

## Supplementary information

Additional file 1: Full list of participants interviewed and type of interview conducted

Additional file 2: Cover Sheet

Additional file 3: Tracking Sheet

Additional file 4: IRISS Messages in Hausa

## Data Availability

All data generated or analyzed during this study are included in this published article and available from the corresponding author on reasonable request.

## Abbreviations

AEFI: Adverse Events Following Immunization
FGD: Focus Group Discussion
IDI: In-depth Interview
IRISS: Immunization Reminder and Information SMS System
KII: Key Informant Interview
LGA: Local Government Area
OPV: Oral Polio Vaccine
PCV: Pneumococcal Conjugate Vaccine
PHC: Primary Health Care
RI: Routine Immunization
WDC: Ward Development Committee
