# Supplementary material for: Designing an SMS reminder intervention to improve vaccination uptake in Northern Nigeria: a qualitative study
Source: BMC Health Serv Res. 2021 Aug 20;21:844. doi: 10.1186/s12913-021-06728-2 (PMC8379866; doi:10.1186/s12913-021-06728-2)
Supplement: Supplementary file 1 — Full list of participants interviewed and type of interview conducted [file 12913_2021_6728_MOESM1_ESM.docx]

**Additional File 1.** Full list of participants interviewed and type of interview conducted

| **Level** | **Category of participants** | **Designation of participants** | **Type of interview** |
| --- | --- | --- | --- |
| State | Policymaker | Executive Secretary Kebbi State Primary Health Care Development Agency | KII |
|  | Program manager | Director, Immunization | KII |
|  |  | Program Manager, Kebbi State Emergency Routine Immunization Coordinating Committee | KII |
|  | Partners | State Lead WHO  State Lead Unicef | KIIs |
| LGA | Program managers | Routine Immunization Officer | KIIs |
| Health facility | Health workers at PHC facility | Officer in charge/Ward focal person, RI In-charge | KIIs |
| Community | Parents | Group of fathers of children under five years of age in two LGAs | FGDs |
|  |  | Group of mothers of children under five years of age in two LGAs | FGDs |
|  |  | Father of children under five years of age | IDI |
|  |  | Mother of children under five years of age | IDI |
|  |  | Mother of new-born | IDIs |
|  | Pregnant women | Pregnant women in two LGAs | IDIs |
|  | Youths | Group of male youths | FGD |
|  |  | Group of female youths | FGD |
|  |  | Young men | IDI |
|  |  | Young women | IDI |
|  | TBAs | TBAs in one LGA | FGD |
|  | WDC | WDC leaders and members in two LGAs | FGDs |
|  | Community/ traditional leaders | Group of opinion leaders (traditional and religious leaders, persons of influence) in two LGAs | FGDs |
|  |  | Opinion leaders (e.g. Imam) | IDIs |
