# Supplementary material for: Designing an SMS reminder intervention to improve vaccination uptake in Northern Nigeria: a qualitative study
Source: BMC Health Serv Res. 2021 Aug 20;21:844. doi: 10.1186/s12913-021-06728-2 (PMC8379866; doi:10.1186/s12913-021-06728-2)
Supplement: Supplementary file 2 — Cover Sheet [file 12913_2021_6728_MOESM2_ESM.docx]

**Additional File 2.** Cover Sheet

**Complete before each interview**

Data collector’s name: ------------------------------------------------------

Note taker’s name: ----------------------------------------------------------

|  | **CATEGORY** |  |  |  |  |  | **CODE** |
| --- | --- | --- | --- | --- | --- | --- | --- |
| **0** | **Interview number** |  |  |  |  |  |  |
|  |  |  |  |  |  |  |  |
| **1** | **Government** |  |  |  |  |  |  |
|  | 1 National |  | 3 State |  | 5 Ward |  |  |
|  | 2 Zone |  | 4 LGA |  | 6 HF |  |  |
| **2** | **Respondent type** |  |  |  |  |  |  |
|  | 1 Elected official |  | 4 Technical officer |  | 7 Community member |  |  |
|  | 2 Director |  | 5 Medical officer |  | 8 Chairperson |  |  |
|  | 3 Assistant Director |  | 6 Health worker |  | 9 Other, please specify |  |  |
| **3** | **Interview date** |  |  |  |  |  |  |
|  | Year 2018 |  | Month |  | Day |  |  |
| **4** | **Interview time** |  |  |  |  |  |  |
|  | Starting time |  | Ending time |  | Total time in minutes |  |  |
| **5** | **Years in position** |  |  |  |  |  |  |
|  |  |  |  |  |  |  |  |
| **6** | **Years in immunization** |  |  |  |  |  |  |
|  |  |  |  |  |  |  |  |
| **7** | **Years in health care** |  |  |  |  |  |  |
|  |  |  |  |  |  |  |  |
| **8** | **Respondent sex** |  |  |  |  |  |  |
|  | 1 Male |  | 2 Female |  |  |  |  |
| **9** | **Respondent age in years** | | |  |  |  |  |
|  |  | | |  |  |  |  |
| **10** | **Respondent Education (completed schooling)** | | |  |  |  |  |
|  | 1 None |  | 2 Primary |  | 4 Graduate |  |  |
|  |  |  | 3 Secondary |  | 5 Post-graduate |  |  |
