# Supplementary material for: Designing an SMS reminder intervention to improve vaccination uptake in Northern Nigeria: a qualitative study
Source: BMC Health Serv Res. 2021 Aug 20;21:844. doi: 10.1186/s12913-021-06728-2 (PMC8379866; doi:10.1186/s12913-021-06728-2)
Supplement: Supplementary file 3 — Tracking Sheet [file 12913_2021_6728_MOESM3_ESM.docx]

**Additional File 3.** Tracking Sheet
