# Supplementary material for: Designing an SMS reminder intervention to improve vaccination uptake in Northern Nigeria: a qualitative study
Source: BMC Health Serv Res. 2021 Aug 20;21:844. doi: 10.1186/s12913-021-06728-2 (PMC8379866; doi:10.1186/s12913-021-06728-2)
Supplement: Supplementary file 4 — IRISS Messages in Hausa [file 12913_2021_6728_MOESM4_ESM.docx]

**Additional File 4.** IRISS Messages in Hausa

| **S/N** | **Normative messages** |
| --- | --- |
| 1 | Kana da masaniyar makwabcin ka ya kai yaransa wurin rigakafi?  Me kake jira? Kada ka bari a barka a baya. |
| 2 | Kana da masaniyar cewa mai martaba sarkin Gwandu na goyon bayan rigakafi?  Kuma yana kira ga mu zama iyaye na gari ta hanyar kai yaranmu wurin rigakafi. |
| 3 | Magidanta muba matan mu dama su kai yaran mu wurin rigakafi. |
| 4 | Malaman mu na addini sunyi ma yaran su rigakafi. To muma me muke jira. |
| 5 | Allah ya dora muna nauyin kula da lafiyar yaran mu.  To mu tabbatar da anyi musu rigakafi. |
| 6 | Ya kamata mu iyaye mu bada lokaci domin kai yaran mu wurin rigakafi.  Domin wannan lokaci ka iya ceton rayuwar yaranmu. |
|  | **Scheduled reminder messages** |
| 7 | Kada ki manta ki adana katin rigakafin yaranki.  Domin zai tuna miki da ranar da zaki sake ziyartar asibitin ….. don amsar rigakafi. |
| 8 | Kada ka/ki manta! Akwai bukatar ziyyartar asibiti sau biyar kafin a kamala rigakafin yaro:  Bayan haihuwa, a sati 6, sati na 10,sati na 14 da kuma wata 9 |
| 9 | za'a gudanar da aikin bada rigakafi kyauta, a fadar Hakimi/Mai Unguwa.  Ki kawo danki/diyarki don ayi musu rigakafi, ki tuna rigakafi na ceton rayuwar yaro. |
| 10 | Asibitin Ribah zata gudanar da aikin bada rigafafi gobe da karfe 8 na safe zuwa karfe 2.  Ka tabbata ka kai danka/diyarka domin yi musu rigakafi.  Ka karfafa ma makwabcin ka domin ya kai yaransa. |
|  | **Motivational messages** |
| 11 | Albishirin ku! Ko kunsan dalilai ukku da ke karfafa muna mukai yaranmu wurin rigakafi.  1) Rigakafi na ceton rayuwa 2) Rigakafi na kare cututtuka 3)Rigakafi bata da illa. |
| 12 | kowa ne ziyara zuwa asibiti domin rigakafi nada matukar amfani.  Ki bada goyon baya domin lafiya mai dorewa.  Ki kasance akan lokaci,kuma ki tabbatar da kin kammala riga kafin yaron ki. |
| 13 | Ki tabbata anyi wa yaron ki/yarki rigakafi koda ta/yayi kuka bayan rigakafin, zasu yi farin ciki idan sun samu lafiya a nan gaba. Kuma zasu tallafa miki. |
| 14 | Iyaye, ba zamu yi farin ciki ba idan yaran mu suka tarbe mu idan muka dawo gida?  Mu faranta musu muma ta hanyar kai su wurin rigakafi. |
| 15 | Ganin yaran mu suna girma cikin koshin lafiya abu ne mai ban sha'wa.  Mutabbatr da anyi musu rigakafi domin ganin haka. |
| 16 | Dukkan rigakafin da ake gudanarwa a asibitin gwamnati kyauta ne.  Tabbas kyautane. Karmu bari wannan danmar ta wuce mu. |
| 17 | Ka goyi bayan rigakafi a duk inda kake.  Ka karfafawa makwabtanka da su kai yaransu domin yi masu ragakafi. |
| 18 | Sanar da jama'ar mu amfanin kiyon lafiya abu ne da ya rataya a wuyan kowa.  Ka bayar da taka gudunmawa ta hanyar sanar da yan'uwa da sauran alumma domin su kaia yaransu wurin rigakafi. |
| 19 | Don tabbatar da kowane yaro anyi masa rigakafi, wajibinmu ne mu yada sakon muhummancin shi ga jama'a. |
| 20 | Taimaka ki yada sakon muhimmancin rigakafi a cikin unguwar ku.  Ki sanar da iyaye cewa rigakafi na ceton lafiyar yara. |
|  | **Educational messages** |
| 21 | Rigakafin farko da yaron ka/ki zai karba sune na ciyan anta,tarin huka da kuma shan inna. Kuma ana yin wanna rigakafi ne a cikin sati biyu da haihuwar yaro. |
| 22 | Rigakafi na biyu da yaron ka/ki zai karba sune na ciyon huhu,shan inna da penta(cutukka guda biyar). Lokacin da yaro ya cika sati 6. |
| 23 | Ko ka taba samun labarin allurar penta? Allura ce da ke kare yaro daga cututuka guda biyar sune ciyon tarin lala,ciyon anta,daji, bugun tsuntsu da tsinkau tsinkau. |
| 24 | Rigakafi na ukku da yaron ka/ki zai karba sune ciyon huhu,shan inna da penta2. Lokacin da ya kai sati 10. |
| 25 | Rigakafi na biyar da yaron ka/ki zai karba sune na ciyon dussa da kuma ciyon shawara lokacin da ya kai wata 9. |
| 26 | Rigakafi na hudu da yaron ka/ki zai karba sune na shan inna,ciyon huhu penta3. Lokacin da yakai sati 14. |
| 27 | Yancin ki ne a baki rigakafi kyauta a kowane asibitin gwamnati. Ki je a ba yaronki rigakafi a asibitin da ke kusa da ke. |
|  | **Informative messages** |
| 28 | Yin zazzabi ko kumburi a inda aka ba da rigakafi ba matsala ba ne. Idan jikin yaro ya kara muni, maza ki kai shi asibiti don a ba shi kulawa. |
| 29 | Rigakafi baya kawo ciwo. Rigakafi yana kare jarirai da kananan yara daga kwayoyin cuta masu kawo ciwo. |
| 30 | Duka rigakafin da ake bayarwa a tsarin rigakafin nijeriya ingantacce ne, kuma gwamnati ta tabbatar da lafiyarsu kafin a ba yara. |
| 31 | Idan yaro yana da zazzabi, ko yana shan maganin antibiotik, za a iya masa rigakafi.  Amma ki tuntubi likitan ka/ki domin karin bayani. |
| 32 | Idan an ba yaro rigakafi fiye da sau daya a lokaci guda, ba zai yi wa yaro illa ba.  Hakan ma, yana kare yaron daga kwayoyin cuta masu sa ciwo. |
| 33 | Idan an ba yaro rigakafi fiye da sau daya a lokaci guda, ba zai yi wa yaro illa ba.  Yana da muhimmanci a kammala dukan rigakafin don a samicikakkar kariya. |
